# Anti-PD-1 therapy for clinical treatment of lymphoma: a single-arm meta-analysis

**DOI:** 10.18632/oncotarget.26223

**Published:** 2018-10-19

**Authors:** Zhe Geng, Yi Xiao, Xiao-Jian Zhu, Cong Ye, Jian-Feng Zhou

**Affiliations:** ^1^ Department of Hematology, Tongji Hospital, Tongji Medical College, Huazhong University of Science and Technology, Wuhan 430030, P. R. China; ^2^ Department of Rheumatology and Immunology, Tongji Hospital, Tongji Medical College, Huazhong University of Science and Technology, Wuhan 430030, P. R. China

**Keywords:** Anti-PD1 antibodies, lymphoma, clinical activity, safety, meta-analysis

## Abstract

Anti-PD1 antibodies exhibit satisfactory efficacy in treating certain types of lymphoma. We conducted this meta-analysis to explore subtypes benefiting from this treatment and the best anti-PD1 therapeutic modalities.

**Methods:**

A quantitative meta-analysis was performed via a systematic search in PubMed, Web of Science, and The Cochrane Library. The pooled overall response rate (ORR), progression-free survival (PFS), complete remission rate (CRR), overall survival (OS) and adverse events (AEs) were calculated and compared. Data were analyzed using a random-effects meta-analysis to determine risk ratios. Heterogeneity across studies was analyzed using Q and I^2^ statistics.

**Results:**

Thirteen articles were selected, and 9 studies were included in the meta-analysis. There was evidence of significant heterogeneity among the studies. According to PD-L1 expression subgroup analysis, the PD-L1-positive group exhibited significantly better outcomes than the PD-L1-negative group (Z=5.481, p=0.000), with pooled ORRs of 0.74 (95% CI: 0.67–0.81) and 0.2 (95% CI: 0.11–0.3), respectively. For PD-L1-positive and PD-L1-negative patients, the pooled CRRs, PFS and OS were 0.21 (95% CI: 0.14–0.29), 0.76 (95% CI: 0.71–0.81) and 1.0 (95% CI: 0.98–1.0) and 0.05 (95% CI: 0.01–0.11), 0.20 (95% CI: 0.09–0.39) and 0.64 (95% CI: 0.45–0.80), respectively; differences were all statistically significant (Z=2.248, p=0.025; Z=3.555, p=0.000; and Z=3.039, p=0.002, respectively). The pooled incidence of treatment-related all-grade AEs and grade-3/4 AEs was 0.84 (95% CI: 0.75–0.92) and 0.21 (95% CI: 0.15–0.29), respectively.

**Conclusion:**

Patients with PD-L1 overexpression in relapsed or refractory lymphoma benefited more from anti-PD-1 therapy. Moreover, treatment with approved PD-1 inhibitors was well tolerated.

## INTRODUCTION

Lymphomas are malignancies of lymphocytes involving malignant cells that are arrested at different stages of differentiation in lymph nodes, bone marrow, and other tissues [1]. According to GLOBOCAN estimates for 2012, the incidence of lymphoma is rising. Indeed, lymphoma accounts for 3–5% of all cancer diagnoses, with approximately 452,000 new cases and 225,000 deaths per year worldwide [2]. Nonetheless, recent advances in molecular genetics have vastly improved our understanding of the biological diversity of this disease and have led to the discovery of novel therapies.

Prior to the mid-1990s, treatment for lymphoma relied on combination cytotoxic chemotherapy, which kills rapidly dividing cells but exposes patients to toxic effects, such as myelosuppression, alopecia, and mucositis [3]. Even in Hodgkin's lymphoma (HL), one of the first cancers to be cured, a combination of chemotherapy and radiotherapy can result in long-term toxicities and thus negatively impact the quality of life of patients [4]. Fortunately, several new classes of molecularly targeted agents with better efficacy and less toxicity have been developed in recent decades; however, these novel agents have varying degrees of efficacy for different types of lymphoma. Among them, the targeting of checkpoint inhibitors, such as programmed death 1 (PD-1) inhibitor, appears to be a promising treatment strategy.

PD-1 is a key immune-checkpoint receptor that is rapidly expressed after T cell activation [5]. PD-1 primarily mediates immunosuppression in peripheral tissues by interacting with PD-1 ligands PD-L1 (B7-H1) and PD-L2 (B7-DC), which are expressed by tumor cells and/or stromal cells. Once PD-1 is engaged by one of these two ligands, it inhibits kinase signaling, which typically leads to T cell activation, thereby suppressing T cell function [1]. PD-L1 is commonly expressed by malignant cells and can interact with PD-1 on T cells to prevent an effective antitumor immune response in the tumor microenvironment. Anti-PD-1 antibodies have been applied for diverse solid tumors, achieving objective and robust responses with an acceptable safety profile [5, 6]. However, lymphomas arise from the immune system itself; thus, the effect of PD-1 blockade within this context is more complex compared with the impact on solid tumors.

Given that most of the clinical trials to date have been designed as non-comparable and single-arm studies, the benefits and safety of anti-PD-1 antibodies in different types of lymphoma remain to be clarified. We therefore conducted this quantitative meta-analysis to evaluate the efficacy and safety of PD-1 blockade for different subtypes of lymphoma.

## RESULTS

### Study inclusion and characteristics

**Figure 1:**
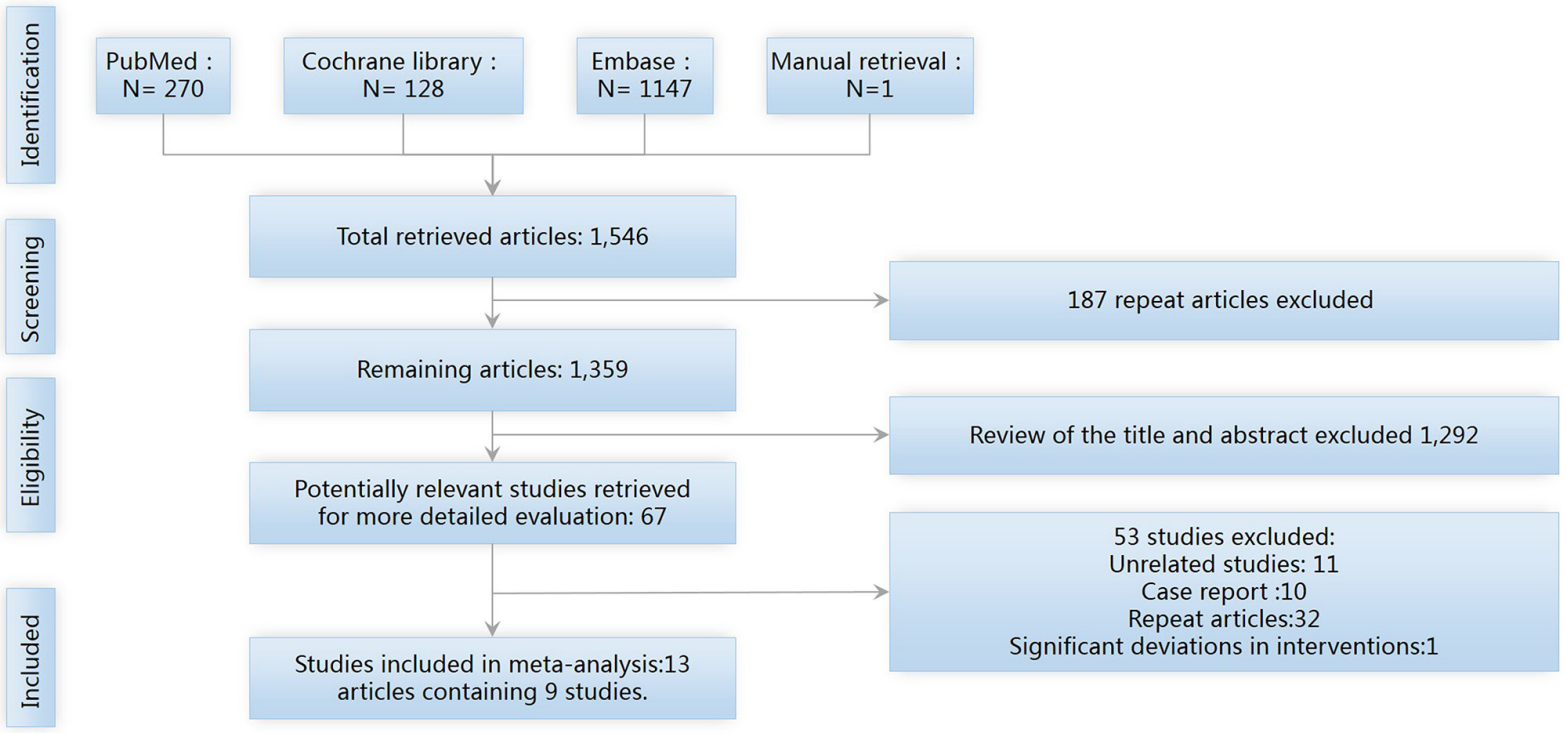
The PRISMA flow diagram of study screening and selection

As shown in Figure 1, our electronic search yielded 1,546 records; one record was manually retrieved. Among the 1,546 articles, EndNote software removed 187 duplicated articles, after which 1,359 articles remained. A review of the title and abstract led to the exclusion of 1,292 unrelated articles, with 66 articles remaining. After carefully reading the full text, 11 unrelated articles were excluded, 10 case reports were excluded, 32 repeat publications were removed, and 1 study was excluded because of significant deviations in interventions. Ultimately, 13 articles on 9 studies were selected [7–19].

### Description of study participants

A total of 665 patients were included in the 13 articles. Nine articles investigated nivolumab, and four articles investigated pembrolizumab. All studies were assessed as fair by the Newcastle-Ottawa Scale (NOS) scoring system. Details of the studies (e.g., registration no., first author, disease type, intervention) are summarized in Table 1.

**Table 1:** Characteristics of the 13 included studies

Registration No.	First author. Year. Ref	N	Disease type	PD-L1/PD-L2	Interventions	NOS score
NCT01592370	Ansell, S. M. 2015 [7]	23	R/R HL	PD-L1:10/10,100%	NV 3 mg/kg Q2 W	5
NCT02572167	Herrera, A. F. 2016 [8]	25	R/R HL	Unclear	(BV 1.8 mg/kg d1, NV 3 mg/kg d8) Q21d	4
NCT02181738	Timmerman, J. M. 2016 [9]	80	cHL received BV after failed ASCT	Unclear	NV 3 mg/kg Q2 W	6
NCT02181738	Zinzani, P. L. 2016 [10]	100	cHL received BV prior to and/or after ASCT	Unclear	NV 3 mg/kg Q2 W	6
NCT01953692	Armand, P. 2016 [11]	31	cHL received BV prior to and/or after ASCT	PD-L1: 15/16, 94% PD-L2: 9/10,90%	Pembrolizumab 10 mg/kg Q2 W	6
NCT01896999	Diefenbach, C. S. 2016 [12]	3 7	R/R cHL	Unclear	NV 3 mg/kg+BV 1.2 mg/kg Q21d NV 3 mg/kg+BV 1.8 mg/kg Q21d	4
NCT01592370	Lesokhin, A. M. 2016 [13]	54	FL (10), DLBCL (11), B-NHL (10), MF (13), PTL (5), T-NHL (5)	PD-L1: (MCL 1;MF1) PD-L2: (B cell NOS 1; MF1)	NV 1 or 3 mg/kg Q2 W	5
JapicCTI-142755	Maruyama, D.2017 [14]	17	R/R cHL (16) ^*3^	Unclear	NV 3 mg/kg Q2 W	6
NCT02857426	Nayak, L.2017 [15]	5^*1^	R/R PCNSL (4), PTL (1)	PD-L1:5/5,100%	NV 3 mg/kg Q2 W	5
NCT02453594	Robert Chen. 2017 [16]	210	R/R cHL (210)	PD-L1:176/177	Pembrolizumab 200 mg Q3 W	6
NCT02332980	Ding, W.2017 [17]	25	R/R CLL (16) RT (9)	0 PD-L1:2/6	Pembrolizumab 200 mg Q3 W	5
NCT01953692	Zinzani, P. L. 2017 [18]	22^*2^	R/R PMBCL	Unclear	Pembrolizumab 10 mg/kg Q2 W	5
NCT02181738	Timmerman, J. M.2017 [19]	63	cHL received BV after ASCT (63)	Unclear	NV 3 mg/kg Q2 W	6

cHL: classical Hodgkin's lymphoma; CLL: chronic lymphocytic leukemia; (R/R) (HL): relapsed or refractory Hodgkin's lymphoma; FL: follicular lymphoma; DLBCL: diffuse large B cell lymphoma; BV: Brentuximab Vedoti; MF: mycosis fungoides, PTL: peripheral T cell lymphoma; RT: Richter transformation; ASCT: autologous stem cell transplant; NV: nivolumab; and PMBCL: primary mediastinal large B cell lymphoma. ^*1^: Inclusion criteria: have tumor tissue for PD-L1 expression testing. ^*2^: The total number of validity data is 20, and the total number of security data is 21. ^*3^: one of these patients was diagnosed with unclassifiable B cell lymphoma, with intermediate features between diffuse large B cell lymphoma (DLBCL) and cHL (intermediate DLBCL/cHL), by a central pathological review committee and was excluded from the efficacy analyses but was included in the safety analyses.

### Clinical activity

#### Objective response rate (ORR)

ORR among 13 articles varied from 16 to 100% (median ORR of 69.15%). A random-effects model determined the presence of significant heterogeneity (I^2^=87.88%, p=0.00). The pooled ORR was 0.68 (95% CI: 0.56–0.80), and there was high, significant heterogeneity regarding outcomes. We further investigated potential sources of heterogeneity by subgroup analyses.

### Subgroup analyses

**Figure 2:**
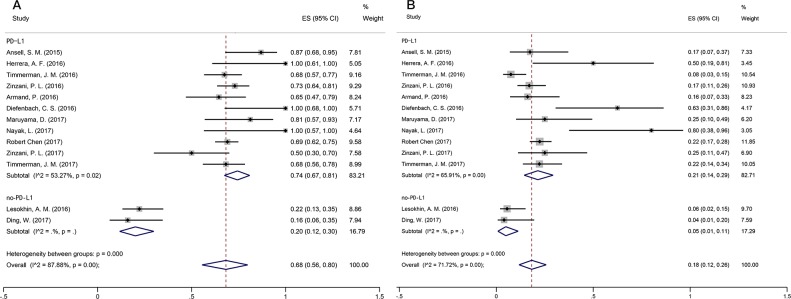
Forest plot showing the result of ORRs and CRRs **(A)** Summary of ORRs for PD-L1-positive and PD-L1-negative patients. **(B)** Summary of CRRs for PD-L1-positive and PD-L1-negative patients.

Basket trials are a new and evolving form of clinical trial design predicated on the hypothesis that the presence of a molecular marker predicts response to a targeted therapy independent of tumor histology. The key strengths of the basket trial design are the ability to identify a favorable response to targeted therapy using a small number of patients and the ability to validate a clinical target. We therefore chose the factor PD-L1 expression for subgroup analyses. The results showed different PD-L1 expression (positive vs. negative, p=0.00) (Figure 2A) (Note: *subgroup criteria are described in the discussion section.*). Eleven articles reported the ORRs of PD-L1-positive lymphoma. The random-effects model was adopted and revealed moderate heterogeneity (I^2^=53.27%, p=0.02). According to the PD-L1 expression subgroup analysis, the pooled ORRs for PD-L1-positive and PD-L1-negative patients were 0.74 (95% CI: 0.67–0.81) and 0.2 (95% CI: 0.12–0.30), respectively, and the difference was statistically significant (Z=5.481, p=0.000) (Table 2). Marked asymmetry was not observed in a funnel plot (Egger's test p=0.866), suggesting the absence of significant publication bias (Table 3).

**Table 2:** Subgroup analysis results

Outcome	Comparison	RRA	RRAuCI	RRAICT	RRB	RRBuCI	RRBICI	Z	*P*
ORR	PD-L1^+^ vs. PD-L1^−^	0.74	0.81	0.67	0.2	0.3	0.12	5.480906	0.0000000423
CRR	PD-L1^+^ vs. PD-L1^−^	0.21	0.29	0.14	0.05	0.11	0.01	2.24479	0.0247816339
PFS	PD-L1^+^ vs. PD-L1^−^	0.76	0.76	0.71	0.2	0.39	0.09	3.554572	0.0003785942
OS	PD-L1^+^ vs. PD-L1^−^	1	1	0.98	0.64	0.8	0.45	3.038716	0.0023758906

**Table 3:** Egger's test for small-study effects

Outcome	Number of studies	*P*
ORR	13	0.866
CRR	13	0.157
All grade TRAEs	12^#^	0.717
Grade 3-4 TRAEs	12^#^	0.944

#: Reference16(Robert Chen(2017)) does not provide detailed informaton about TRAEs.

#### Complete remission rate (CRR)

CRR varied from 4 to 80% (median CRR was 27.3%) across the studies. The overall CRR was 0.18 (95% CI: 0.12–0.26), with significant heterogeneity (I^2^=71.72%, p=0.00). In our subgroup analysis on PD-L1-positive and -negative lymphoma, a random-effects model showed heterogeneity (I^2^=65.91%, p=0.00) (Figure 2B). The pooled CRRs for PD-L1-positive and PD-L1-negative patients were 0.21 (95% CI: 0.14–0.29) and 0.05 (95% CI: 0.01–0.11), respectively, and the difference was significant (Z=2.245, p=0.025) (Table 2). Marked asymmetry was not observed in a funnel plot (Egger's test p=0.157), indicating a lack of significant publication bias (Table 3).

#### Progression-free survival (PFS)

**Figure 3:**
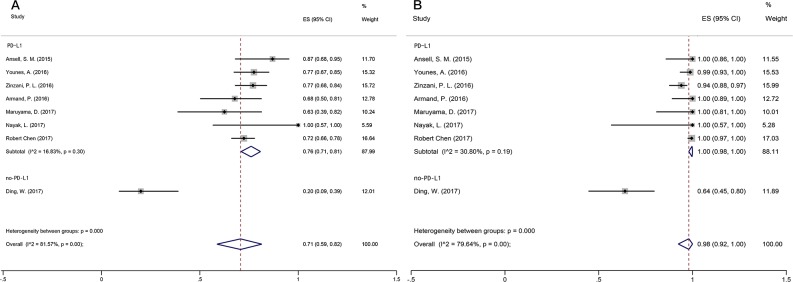
Forest plot showing the result of PFS and OS **(A)** Summary of PFS for PD-L1-positive and PD-L1-negative patients. **(B)** Summary of OS for PD-L1-positive and PD-L1-negative patients.

Among the studies, PFS varied from 20 to 100% (median PFS of 70.5%). Overall 6-month PFS was 0.71 (95% CI: 0.59–0.82), with significant heterogeneity (I^2^=81.57%, p=0.00). A random-effects model was adopted for subgroup analysis on PD-L1-positive and -negative lymphoma, showing acceptable heterogeneity (I^2^=16.83%, p=0.30) (Figure 3A). The pooled PFS for PD-L1-positive and PD-L1-negative patients was 0.76 (95% CI: 0.71–0.81) and 0.20 (95% CI: 0.09–0.39) (Table 2), respectively, and these values differed significantly (Z=3.555, p=0.000) (Table 2).

#### Overall survival (OS)

OS across the studies varied from 64 to 100% (median OS was 94.62%). Overall OS was 0.98 (95% CI: 0.91–1), with significant heterogeneity (I^2^=79.64%, p=0.00). We performed another subgroup analysis on PD-L1 positive and PD-L1-negative lymphoma. Again, a random-effects model was adopted and revealed acceptable heterogeneity (I^2^=30.80%, p=0.19) (Figure 3B).

The pooled OS was 0.76 (95% CI: 0.71–0.81) for PD-L1-positive and 0.2 (95% CI: 0.09–0.39) for PD-L1-negative patients, which was a significant difference (Z=3.039, p=0.002) (Table 2).

### Safety (Table 4)

**Table 4:** Drug-related adverse events

First author. Year. Ref	Follow up months	All grade	G3-4	Most common AE	SAEs	IrAEs	Discontinue treatment#
Ansell, S. M. 2015 [7]	10 (0 to 18.75).	78%	22%	Rash 22%, thrombocytopenia 17%	Pancreatitis1; MDS1; lymph-node pain 1	Unclear	MDS and thrombocytopenia 1; And pancreatitis 1
Herrera, A. F. 2016 [8]	Unclear	78%	13%	Fatigue 35%, nausea 26%, rash 22%, dyspnea 17%, myalgia17%, and pruritus 17%	(Dehydration, hypercalcemia, and acute kidney injury) 1	Rash 2; and hypothyroidism 1	0
Timmerman, J. M. 2016 [9]	15.4 (1.9-18.5)	93%	29%	Fatigue 11%, infusion reaction 11%, and diarrhea 11%	Pyrexia, pneumonia, tumor progression, arrhythmia, infusion reaction, and meningitis (≤4% each)	Unclear	Unclear
Zinzani, P. L. 2016 [10]	8.8	68%	19%	Pyrexia 13%, diarrhea 11%, cough 8%, fatigue 8%, and neutropenia 8%	Unclear	Unclear	0
Armand, P. 2016 [11]	24.9 (7.0-29.7)	97%	16%	Hypothyroidism 16%, diarrhea 16%, nausea 13%, and pneumonitis 10%	Colitis 1; increased ALT and AST levels 1; axillary pain 1; back pain and nephrotic syndrome 1; and joint swelling 1	Unclear	G2 pneumonitis 1 G3 nephrotic syndrome 1
Diefenbach, C. S. 2016 [12]	3.6	90%	20%	Transaminitis 29%, peripheral sensory neuropathy19%, and rash 9.6%	0	Unclear	(Pneumonitis G3 with G3 dyspnea, hypoxia, and typhilits G3) 1
Lesokhin, A. M. 2016 [13]	16.65 (0.4-33.0)	72%	24%	Skin (pruritus, rash) 18%, fatigue 17%, pneumonitis 11%, and decreased appetite 9%	G5 (fatal pneumonitis/ARDS) 1	G1 or G2 28 (only 15 required treatment; of these, five had to discontinue nivolumab)	G1 (myositis and conjunctivitis) 1; G2 (enteritis and pneumonitis) 2; G3 (pneumonitis, stomatitis, neutropenia, diplopia, creatine phosphokinase increase, and rash) 6; G4 (pneumonitis, pustular rash, and sepsis) 3
Maruyama, D.2017 [14]	9.8 (6.0-11.1)	100%	23.50%	Pyrexia 41.2%, pruritus 35.3%, rash 35.3%, and hypothyroidism 29.4%	(Pyrexia, hepatic function abnormal, hyponatremia, fulminant type 1 diabetes mellitus, interstitial lung disease and rash) 3	Skin disorders 8;Endocrine disorders 6 Gastrointestinal disorders 3 Hepatic disorders 2 Pulmonary disorders 1 Hypersensitivity and infusion reactions 1	Interstitial lung disease 1; rash 1; and G2 peripheral neuropathy 1
Nayak, L.2017 [15]	median 17	40%	0%	Unclear	0	0	0
Robert Chen. 2017 [16]	10.1 (1.0-15.0)	Unclear	Unclear	Hypothyroidism 12.4%, pyrexia 10.5%	0	Unclear	9 (Myocarditis, myelitis, myositis, pneumonitis, infusion-related reactions, and cytokine release syndrome)
Ding, W.2017 [17]	10.4 (2.7-16.1)	100%	60%	Cough 28%, thrombocytopenia 24%, anemia 20%, nausea 20%, neutropenia 16%, dyspnea 16%, fatigue 12%, diarrhea 12%, and vomiting 12%	1 G3 lung infections 1 G3 hepatic toxicities 2 G2 pneumonitis 2 Early death	Unclear	Unclear
Zinzani, P. L. 2017 [18]	14.3 (0.6-34.7)	67%	23.8%	Unclear	7	Unclear	0
Timmerman, J. M.2017 [19]	14 (1-20)	75%	11%	Fatigue 29% and diarrhea 21%	Unclear	Unclear	Unclear

SAE: Drug-related serious adverse events; IrAEs: immune-related adverse events; MDS: myelodysplastic syndrome; and discontinued treatment: patients had toxic effects.

**Figure 4:**
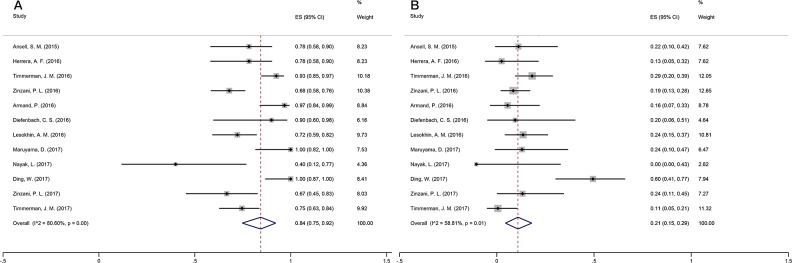
Forest plot showing the result of TRAEs **(A)** ALL Grade TRAEs. **(B)** Grade 3-4 TRAEs.

The common treatment-related adverse events (TRAEs) included fatigue, rash, diarrhea, pruritus, decreased appetite, and nausea. The number of all-grade AEs and grade-3/4 AEs was available in 12 studies. Regarding the absolute risk of AEs, the heterogeneity test results (I^2^=80.60%, p=0.00) indicated significant heterogeneity among the studies. Therefore, the random-effects model was employed to combine effects. The incidence of PD-1 antibody-associated AEs was 0.84 (95% CI: 0.75–0.92) (Figure 4A).

Regarding grade-3-4 AEs, the results of the heterogeneity test (I^2^=58.81%, p=0.01) indicated significant heterogeneity. As a result, the random-effects model was used to combine effects. The incidence of PD-1 antibody-associated grade-3/4 AEs was 0.21 (95% CI: 0.15–0.29) (Figure 4B).

#### Treatment-related serious adverse events (SAEs)

In published trials on PD-1 inhibitors, the most common SAEs included the following: digestive (hepatotoxicity, pancreatitis, colitis, and duodenitis); respiratory (pneumonia); urinary (acute kidney injury and nephrotic syndrome); nervous (suppurative meningitis); cardiovascular (arrhythmia); and other (fever, axillary pain, and infusion reaction).

#### Immune-related adverse events (IrAEs)

In published trials on PD-1 inhibitors, the most common IrAEs were as follows: skin disorders (rash); endocrine disorders (hypothyroidism); gastrointestinal disorders; hepatic disorders; pulmonary disorders; hypersensitivity; and infusion reactions.

#### Patients discontinued treatment for the following reasons related to drug toxicity

myocarditis, myelitis, myositis, pneumonitis, infusion-related reactions, cytokine release syndrome, MDS, thrombocytopenia, and pancreatitis.

### Publication bias (Table 3)

**Figure 5:**
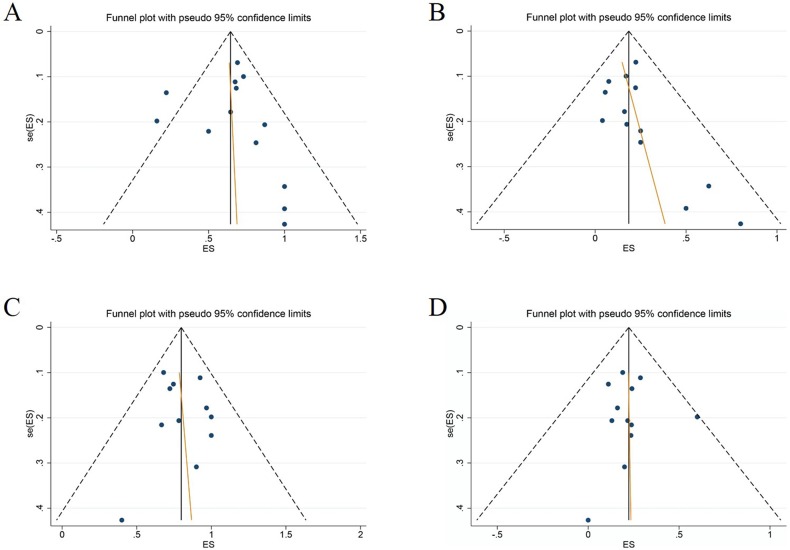
Funnel plots for **(A)** ORR; **(B)** CRR, **(C)** All grade TRAEs, and **(D)** Grade 3-4 TRAEs.

The funnel plots generated were inspected for geometry and found to be symmetrical, suggesting the absence of publication bias (Figure 5), which was statistically confirmed by Egger's test (ORR, p=0.866; CRR, p=0.157; all-grade TRAEs, p=0.717; grade-3-4 TRAEs, p=0.944). Publication bias, as determined by the Begg test, was not statistically significant for any of these outcomes.

## DISCUSSION

In this meta-analysis, anti-PD-1 antibodies were found to have promising clinical activity in certain types of lymphoma, particularly in PD-L1-positive patients. The median ORR was as high as 69.15% for the 13 included studies, which was higher than that of solid-tumor patients reported by Zhang et al. [20]. In a recent meta-analysis, Zhang and colleagues confirmed the efficacy of antiPD-1/PD-L1 antibodies for various cancers, especially melanoma, non-small-cell lung cancer (NSCLC) and renal cell carcinoma (RCC) [20]. Nonetheless, the ORRs were only 29% (95% CI: 1.53−2.41), 21% (95% CI: 17%−25%) and 21% (95% CI: 16%−27%), respectively. These low percentages may have resulted from insufficient T cell infiltration and the highly immunosuppressive microenvironment of solid tumors [20].

Substantial heterogeneity was expected in the current meta-analysis because of the well-known heterogeneity among lymphomas. The ORRs across the 13 studies included varied from 16 to 100%. In addition, heterogeneity was prominent between the trials (I2=87.88%, p=0.00). According to previous reports of other cancers, tumor PD-L1 protein expression is related to improved benefits and better outcomes in patients treated with anti-PD-1 monoclonal antibodies [21, 22]. Accordingly, we further analyzed the included studies by categorizing the patients as PD-L1 positive or negative. Among the 13 studies, several evaluated PD-L1 expression in the tumor by immunohistochemistry (IHC), whereas positive PD-L1 expression in tumor cells was an inclusion criterion for selection in other studies. Although some studies did not evaluate PD-L1 expression in the tumor, we can still expect high PD-L1 expression in the patients included, as these studies enrolled cases of HL and primary mediastinal large B cell lymphoma (PMBCL). It is well characterized that in more than 85% of classic HLs, tumor (Hodgkin's/Reed Sternberg) cells overexpress PD-L1 and PD-L2 due to a genetic mutation in 9p24, as observed in primary mediastinal B cell lymphoma [23]. Moreover, this mutation results in increased copy numbers of the PD-L1 and PD-L2 genes. Consistent with these observations, subgroup analysis revealed a significant correlation between ORR and PD-L1 expression.

The pooled ORRs were 0.2 (95% CI: 0.11–0.3) and 0.74 (95% CI: 0.67–0.81), respectively, for PD-L1-negative and PD-L1-positive patients, and this difference was statistically significant (Z=5.481, p=0.000) (RR=2.08; 95% CI: 1.49–2.91; p<0.01). Similarly, CRR, PFS, and OS were better in PD-L1-positive patients, which further supports the notion that PD-L1 overexpression is related to a better treatment response from anti-PD-1 therapy.

Although several clinical trials on PD-1 pathway-blocking agents, administered alone or in combination with other therapies, have reported very encouraging results, dramatic responses are not observed in all lymphoma patients treated with PD-1-blockade therapy [24]. Indeed, Lopes and colleagues have emphasized the importance of biomarkers for novel therapies: by reviewing more than 10,000 trials involving 1,079 drugs, the success rate of drugs developed with biomarkers increased from 6 to 24% compared with drugs developed without biomarkers. In addition, in a mouse model of human T cell non-Hodgkin's lymphoma, Wartewig et al. showed that treatment of the mice with a PD-1 antibody, as would be performed for patients, led to rapid and lethal proliferation of cancerous T cells [25]. Such findings highlight the importance of selecting proper candidates for PD-1-blockade therapy. Therefore, predictive biomarkers are necessary to improve the development of anti-PD-1 treatment for lymphoma.

It is expected that tumor expression of PD-L1 may be a predictive biomarker because it reflects protein expression levels of the target of anti-PD-1 agents, and effective blocking action requires pre-existing PD-L1−PD-1 interaction in tumors. It is well established that overexpression of PD-L1 in tumor cells facilitates cancer immune evasion by inhibiting cytotoxic T cell functions [26, 27]. Therefore, elevated PD-L1 expression in tumors should correlate with a poor prognosis and with an improved therapeutic effect due to PD-1 blockade. In fact, PD-L1 expression has been used as an effective prognostic and/or predictive biomarker for certain solid tumors [28, 29]. The PD-1 antibodies already approved by the US Food and Drug Administration (FDA) include nivolumab (OPDIVO; Bristol-Myers Squibb Co.) and pembrolizumab (KEYTRUDA; Merck & Co., Inc), though the latter was only approved for patients whose tumors express PD-L1 [30]. Moreover, a meta-analysis of anti-PD-1/PD-L1 antibodies for treatment of advanced or refractory cancers concluded that tumor PD-L1 expression and patient smoking status might serve as biomarkers to predict response to anti-PD-1/PD-L1 antibody treatment, especially for patients with melanoma, non-small cell lung cancer (NSCLC) and renal cell carcinoma (RCC) [20]. In addition, another meta-analysis by Gandini et al. also demonstrated that PD-L1 expression is significantly associated with mortality and clinical response to anti-PD-1/PD-L1 antibodies in metastatic melanoma patients and with clinical response in patients with non-squamous NSCLC [31]. However, this is not the case for all neoplasms, such as Basal-like breast cancer and colorectal cancer [32, 33]. These contradictory findings suggest that the predictive value of tumor PD-L1 expression as a biomarker may depend on the type of tumor. The complexity of the immune signaling network as well as dynamic and clustered PD-L1 expression patterns may also contribute to varying responses. In our meta-analysis, we found tumor PD-L1 expression to be a robust prognostic factor for the general effect of PD-1 blockade in lymphoma, which is consistent with the findings of a previous network meta-analysis on advanced NSCLC [21]. Additionally, testing of PD-1/PD-L1 inhibitors in early-phase trials has been accompanied by the parallel development of companion diagnostic assays with which to evaluate PD-L1 immunohistochemical staining of immune cells and/or certain tumor cells, such as NSCLC and squamous cell carcinoma of the head and neck (SCCHN) [34]. Despite the uncertainty, the FDA has approved two companion diagnostic PD-L1 IHC assays [35].

Of note, assessment of PD-L1 tumor expression is currently a controversial issue. First, several studies have hypothesized that expression of PD-L1 on tumor cells can be a dynamic process that varies based on different tumor microenvironmental stimuli and can be difficult to evaluate using a single, small paraffin-embedded tissue sample [36]. Second, there is no standard definition to date of the exact cut-off value considered to indicate overexpression [37]. Despite these drawbacks, we still believe that determination of PD-L1 overexpression by IHC in lymphoma may predict a better response to anti-PD-1 treatment.

Based on our meta-analysis, anti-PD-1 antibodies are well tolerated among lymphoma patients. The most common AEs are fatigue, rash, pruritus, nausea, pyrexia, hypothyroidism, diarrhea and abnormal hepatic function, which are consistent with those reported by another meta-analysis on solid tumors [38]. Furthermore, immune-related AEs (e.g., abnormal hepatic function, hypothyroidism) were observed in only a small proportion of patients. Most AEs were of grade 1 or 2 and were shown to be manageable. Documented SAEs included pneumonitis, hepatic toxicity, acute kidney failure, duodenitis, pancreatitis, fulminant type 1 diabetes mellitus and interstitial lung disease, though their frequencies were low. There were two cases of fatal TRAEs, both of which were pneumonitis. One occurred in a small lymphocytic B cell lymphoma patient [13] and the other in a chronic lymphocytic leukemia (CLL) patient [17]. Patients with these types of lymphoma are usually at increased risk of developing infectious complications, and the risk is further increased in those receiving treatment for the disease [39]. Furthermore, CLL is often associated with autoimmune manifestations [39], which may explain why most of the immune-related AEs were reported in a study that enrolled patients with this subtype of lymphoma [17]. According to this investigation, PD-1 inhibitor treatment is not an optimal choice for patients with CLL.

Similar to any study, ours has limitations. A major limitation may be considered the relatively small sample size of studies included in our meta-analysis. Because immunotherapy with PD-L1 blockade has not been used in lymphoma until recently, there were limited phase I or phase IB single-arm trials available for our study. Another limitation is the absence of grade-3-4 AE data in one of the 13 studies included in the safety analysis (Table 3). Despite these drawbacks, we believe that the results of our work are sufficient to address the main question of this study and have important clinical implications. The findings will provide practitioners with at least some clues for identifying candidates for such treatment and may help in disease-management decisions. Regardless, more and larger studies are warranted in the future to improve our knowledge of the efficacy of PD-L1-blockade therapy among patients with different subtypes of lymphoma.

In conclusion, the results of our meta-analysis indicate that PD-1 pathway-blocking agents are a promising novel therapy for lymphoma and that PD-L1 may be a candidate molecular marker for the identification of patients who may benefit the most from PD-1-blockade therapy. PD-L1 expression on the surface of tumor cells, as shown by pathological IHC, is usually associated with a better response to PD-1-blockade therapy. Furthermore, the approved PD-1 antibodies are well tolerated and are associated with a low risk of severe treatment-related AEs.

## MATERIALS AND METHODS

We searched PubMed, Web of Science, and The Cochrane Library for articles published until July 15^th^, 2017. We reviewed records at the American Society of Hematology (ASH), American Society of Clinical Oncology (ASCO), and European Society for Medical Oncology (ESMO). We also manually retrieved articles on special lymphomas, such as hairy cell leukemia, lymphomatoid granulomatosis, hydroa vacciniforme-like lymphoproliferative disorder, indolent T cell lymphoproliferative disorder of the gastrointestinal (GI) tract, mycosis fungoides, Sezary syndrome, primary cutaneous CD30-positive T cell lymphoproliferative disorders, and lymphomatoid papulosis. To increase sensitivity, the search strategy used both MeSH terms and free-text words. To maximize search sensitivity, no filters or limits on language were applied (Retrieval process charts 1, 2, and 3). The study was approved by the Ethics Committee of Tong Ji Hospital.

### Literature screening and data extraction

The inclusion criteria were as follows: (1) articles investigating the use of anti-PD-1 antibodies on lymphoma patients; and (2) studies reporting any of the following information - ORR, CRR, PFS, OS, and TRAEs.

The exclusion criteria were as follows: (1) repeat articles, letters, editorials, expert opinions, case reports and reviews; (2) studies without usable data; and (3) studies with great heterogeneity.

### Data extraction

Two investigators independently extracted data from eligible studies, and disagreements were resolved by discussion with a third investigator. For each study, the following information was recorded: basic information (e.g., first author, year of publication), research characteristics (e.g., study phase, type of anti-PD-1 inhibitor, drug dose and usage, course of treatment), study subject characteristics (e.g., disease type, number, gender, age, risk rating) and outcome indicators (e.g., ORR, CRR, PFS, OS, and TRAEs).

### Quality control

All of the included studies were single-arm studies. Therefore, we used the NOS to assess quality [40]. Assessment scores of 0–3, 4–6, and 7–9 indicated poor, fair, and good studies, respectively. Discrepancies were resolved by consensus.

### Publication bias

Funnel plots can be used to detect publication bias, but they require a range of studies of varying sizes and subjective judgments. Publication bias was analyzed using Egger's linear regression test, which measures funnel plot asymmetry on a natural logarithmic scale of ORs.

### Statistical analysis

The pooled ORR, CRR, PFS, and OS were analyzed using Stata14.0 (4905 Lakeway Drive College Station, Texas 77845-4512 USA). Data were analyzed by random-effects meta-analyses to obtain risk ratios. Heterogeneity of the data was evaluated by the chi-square Q test and I^2^ statistic. For the Q test, a p value less than 0.1 indicated significant heterogeneity; for I^2^ statistics, an I^2^ value greater than 50% indicated significant heterogeneity. If there was significant heterogeneity among multiple studies after merging the statistical results, subgroup analyses were performed to investigate the reasons for the observed heterogeneity.

The following formulas were used to compare the different studies. Statistical significance was defined as a p value less than 0.05.

$$\begin{array}{l} Z=\frac{{\text{lnRR}}_{A}-{\text{lnRR}}_{B}}{\sqrt{\text{variance of }{\text{lnRR}}_{A}+\text{variance of }{\text{lnRR}}_{B}}};\\ \text{variance of lnRR}={\left[\frac{\ln(\text{upper }\text{CI})-\ln(\text{lower }\text{CI})}{2\times Z\text{ score }\text{for}\text{ upper }\text{CI}\text{ boundary}}\right]}^{2} \end{array}$$
